# Functional Alignment Philosophy in Total Knee Arthroplasty – Rationale and technique for the varus morphotype using a CT based robotic platform and individualized planning

**DOI:** 10.1051/sicotj/2022010

**Published:** 2022-04-01

**Authors:** Jobe Shatrov, Cécile Battelier, Elliot Sappey-Marinier, Stanislas Gunst, Elvire Servien, Sebastien Lustig

**Affiliations:** 1 Department of Orthopaedics, Croix Rousse Hospital, University of Lyon 1 69004 Lyon France; 2 Sydney Orthopaedic Research Institute Chatswood 2065 Australia; 3 Univ Lyon, Claude Bernard Lyon 1 University, IFSTTAR, LBMC UMR_T9406 69622 Lyon France

**Keywords:** Alignment philosophy, Knee alignment, Functional alignment, Total knee arthroplasty, Robotically assisted arthroplasty

## Abstract

*Introduction*: Alignment techniques in total knee arthroplasty (TKA) continue to evolve. Functional alignment (FA) is a novel technique that utilizes robotic tools to deliver TKA with the aim of respecting individual anatomical variations. The purpose of this paper is to describe the rationale and technique of FA in the varus morphotype with the use of a robotic platform. *Rationale*: FA reproduces constitutional knee anatomy within quantifiable target ranges. The principles are founded on a comprehensive assessment and understanding of individual anatomical variations with the aim of delivering personalized TKA. The principles are functional pre-operative planning, reconstitution of native coronal alignment, restoration of dynamic sagittal alignment within 5° of neutral, maintenance of joint-line-obliquity and height, implant sizing to match anatomy and a joint that is balanced in flexion and extension through manipulation of implant positioning rather than soft tissue releases. *Technique*: An individualized plan is created from pre-operative imaging. Next, a reproducible and quantifiable method of soft tissue laxity assessment is performed in extension and flexion that accounts for individual variation in soft tissue laxity. A dynamic virtual 3D model of the joint and implant position that can be manipulated in all three planes is modified to achieve target gap measurements while maintaining the joint line phenotype and a final limb position within a defined coronal and sagittal range. *Conclusion*: Functional alignment is a novel knee arthroplasty technique that aims to restore constitutional bony alignment and balance the laxity of the soft tissues by placing and sizing implants in a manner that it respects the variations in individual anatomy. This paper presents the approach for the varus morphotype.

## Introduction

The alignment goal in total knee arthroplasty (TKA) continues to be debated. Alignment philosophy’s have evolved since the 1980’s when Insall et al. first described mechanical alignment (MA) as a technique for TKA [1]. While offering satisfactory implant survival, approximately 10–20% of patients are not satisfied following TKA [2, 3], and 33–54% report ongoing symptoms or functional problems [2].

Alternative alignment philosophies have been proposed to restore knee kinematics, believing this will improve clinical outcomes and reduce patient dissatisfaction [4–6]. Hungerford and Krackow first proposed an anatomic alignment (AA) in 1985 that aimed to restore the joint line obliquity while maintaining a MA [7]. Kinematic alignment (KA) philosophy was developed by Howell et al. in 2008 [5] and aimed to restore the kinematics of the native pre-osteoarthritic knee [8]. Concern regarding complications associated with extreme alignment outliers led to developing a restricted kinematic alignment (rKA) by Almaawi et al., which aims to reconstitute native alignment within ±3° of a neutral alignment [6]. Furthermore, alignment techniques to date have described the same approach to varus and valgus morphotypes despite significant differences in anatomy and behavior. Recently it was demonstrated that a rigid approach to aspects of component positioning such as femoral rotation fail to account for the wide variation of anatomy observed and lead to unbalanced compartments, particularly in flexion [9].

With the subsequent arrival of new technologies such as robotic platforms, alignment can be better tailored to patients’ individual bony anatomy and native ligament balancing. A major advancement of some robotically assisted platforms is 3D pre-operative planning with the use of imaging modalities such as CT scans that allow for an assessment of the bony anatomy in all three planes to tailor implant positioning and sizing. While the accuracy and optimal technique of laxity measurements are still to be determined, these assistive technologies allow a quantifiable intraoperative assessment of soft tissue balancing, representing a major advancement from the previous method. Indeed, the results of knee arthroplasty are affected not only by alignment but also soft-tissue balance the restoration of joint line height and obliquity (JLO) [10–13]. The robotic-assisted systems improve implant sizing and positioning [14, 15] and offer the ability for precise adjustments to be made to achieve the desired gap target, joint line-height, and orientation [16, 17]. Functional alignment is an emerging philosophy that aims to reconstruct 3-dimensional constitutional alignment while respecting the behavior of the soft tissue envelope with the assistance of a robotic platform. While its concept has been described [18, 19], rationale and technique are yet to be defined.

The purpose of this paper is to describe the rationale and our technique of functional alignment for the varus morphotype using an image-based robotically assisted platform and pre-operative individualized planning.

### I. Functional alignment principles

#### a. Individualized pre-operative planning

**Figure 1:**
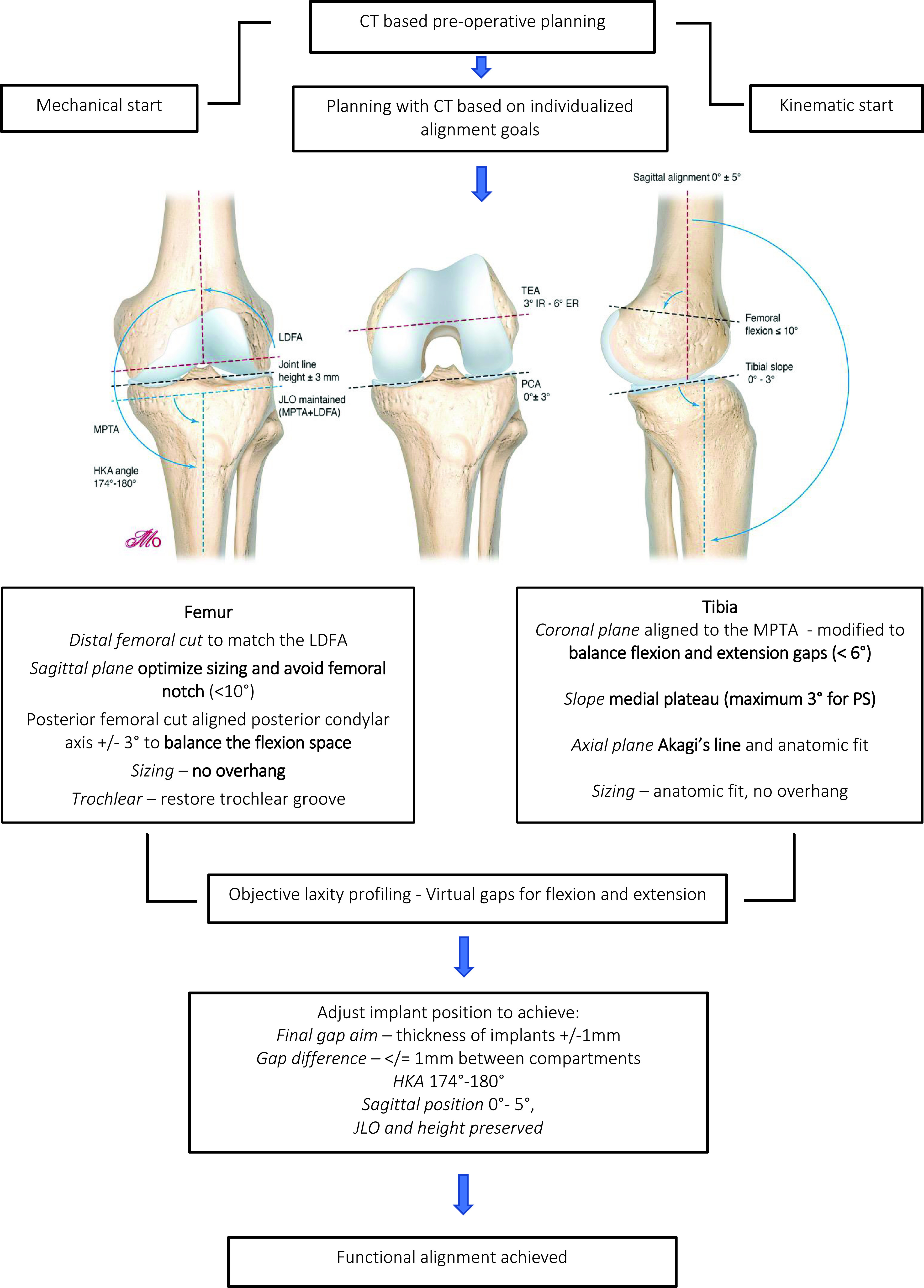
Functional alignment surgical workflow.

**Figure 2:**
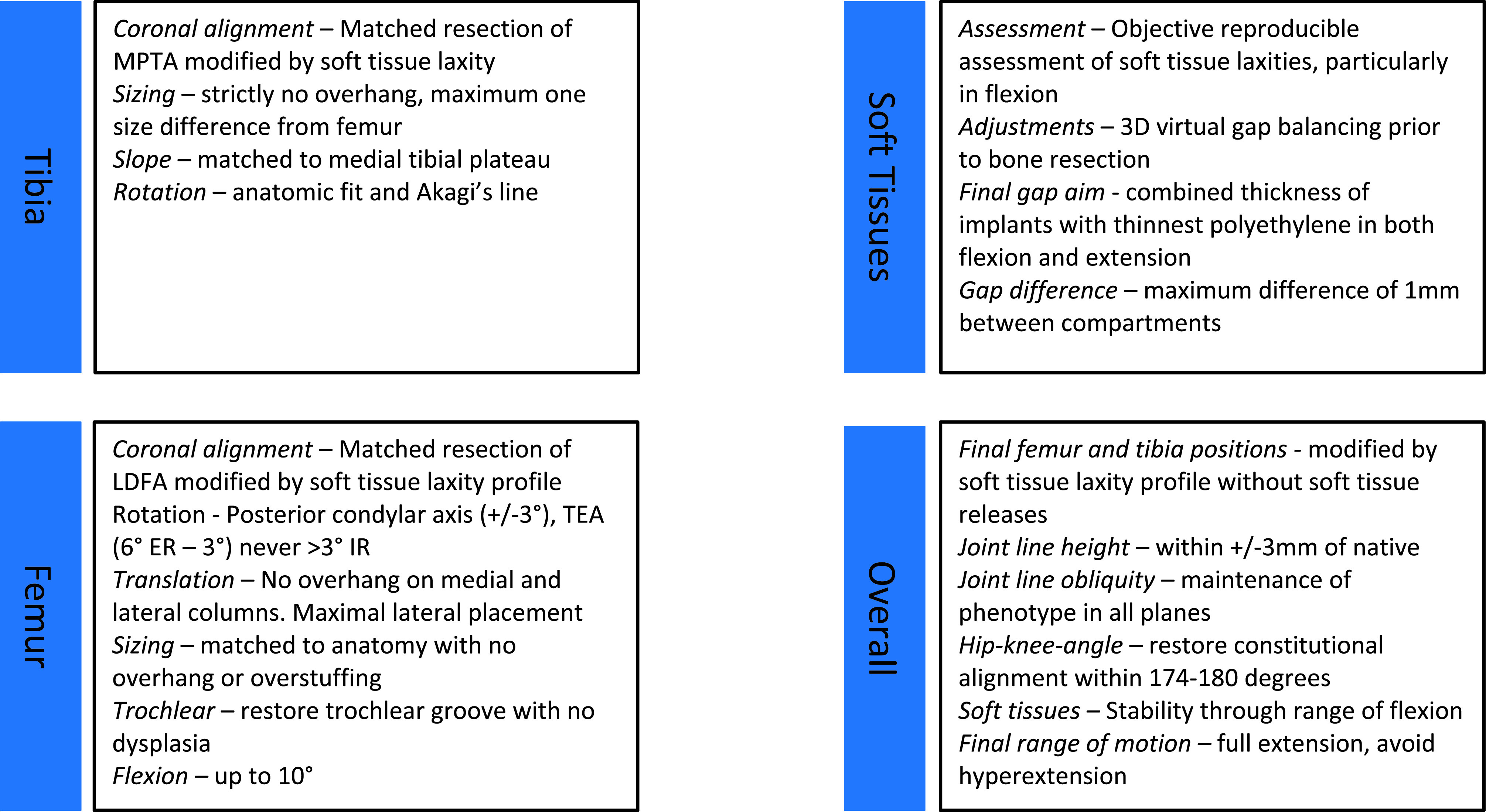
Functional Alignment Principals.

Functional alignment begins with 3D imaging to create a personalized plan based on individual anatomy (Figure 1) and within set boundaries (Table 1). A summary of principles is presented in Figure 2.

**Table 1:** Functional Alignment Philosophy Protocol Guidelines for the varus morphotype.

Parameter	Target
Final coronal alignment (HKA)	174°–180°
Final sagittal alignment with gravity only	0° ± 5°
Femur	
Varus/valgus*	3° varus to 6° valgus
Flexion*	0°–10°
Rotation (PCA)	0° IR–6°ER
Transepicondylar axis (TEA)	3° IR–6° ER
Tibia	
Varus/valgus*	0°–6° varus
Slope*	0°–3° (depends if CR/CS or PS)
Rotation	Manual
	Combination of Akagi’s line, anatomic fit and floating method
JLO and height	JLO orientation to not be changed to different phenotype (CPAK)
	Final joint line height ±3 mm from native
Implant sizing	Femur – matched to curvature of distal femoral radius to avoid notching and medial-lateral condyles to avoid any overhang or over-stuffing
	Tibia – maximal cortical contact with correct rotation with no overhang
	Downsized if there is any conflict
Balancing	Gaps to match the global thickness of the implant at: 0° extension 90° flexion
	Maximum gap difference 1 mm between medial and lateral compartments with a slight lateral laxity acceptable

_*Combined values between tibia and femur more important than isolated values. Individual manufacturing guides may vary between implants._

A significant difference of 3D-based imaging modalities to 2D modalities is the ability *to assess the axial plane, allowing for a more detailed plan of the femoral and tibial rotation* before the surgery. Furthermore, potential problems with implant position or sizing due to anatomic variations or abnormalities can be anticipated. In our experience, this occurs in 10% of cases. The CT image with the virtual 3D implant position and associated bony cuts are reviewed along with plain X-rays. FA creates an individualized plan using the following steps:

- *Femur*
  1. Size of femoral implant: selected using posterior referencing with the smallest size that does *not overhang, notch the anterior femur, or overhang mediolateral bone edges,* and avoids overstuffing the patellofemoral joint.
  2. Coronal plane: femoral implant positioning is modified from a starting point of 0° to the mechanical axis to match the lateral distal femoral angle (LDFA) to aim for constitutional alignment and preserve the JLO.
  3. Sagittal plane: femoral component is positioned to *optimize the component sizing by matching the concentricity of Blumensaats line in cases without trochlea dysplasia and to avoid femoral notching.*
  4. Axial plane: femoral component is aligned to the trochlear groove and posterior condylar axis within 3° of freedom to *balance the flexion gap.*
  5. Resection depth: 9 mm on both medial and lateral distal femoral condyles and both medial and lateral posterior femoral condyles. The target of 9 mm resection is based on 7 mm bone plus 2 mm cartilage.
  6. *Tibia*

1. Coronal plane: tibial implant position is aligned to match the medial proximal tibial axis (MPTA) and *balance flexion and extension gaps by up to 6° of varus*. Valgus tibial position should be avoided. phrase. Previously we found changing a tibia with constitutional varus to a valgus position was associated with an increased risk of revision [20].
2. Sagittal plane: tibial implant position is set to *match the patient’s native posterior tibial slope with a limit of 3° when using a PS implant* and can be modified to balance the flexion gap if necessary. This is adapted to avoid more than 10° of combined femoral-tibial flexion.
3. Axial plane: tibial implant is positioned using *Akagi’s line* and the floating technique.
4. Tibial resection: 8 mm resection (6 mm bone + 2 mm cartilage) is set with a maximum 6° varus based on subchondral bone and an average cartilage depth of 2 mm in the normal knee [11], to use a 9 mm polyethylene insert. This gives a combined planned resection of 17 mm, which is the combined thickness of this implant with its thinnest tibial liner (Figure 3).
  **Figure 3:**
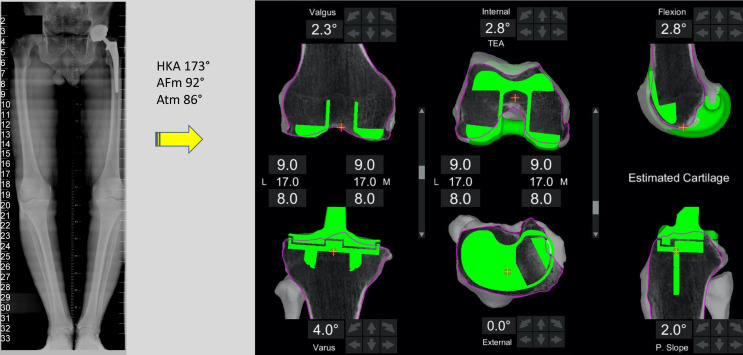
Typical plan for a varus morphotype knee.
5. *Hip-knee-angle*

If the HKA angle (calculated as the sum of the femoral coronal alignment and the tibial coronal alignment [12]) falls outside of 6° varus to 0° valgus for a varus knee, alterations are made to the implant that contributes most to the deformity based on the MPTA and LDFA. A typical plan for a varus knee is presented in Figure 3.

#### b. Coronal alignment to aim for constitutional alignment

**Figure 4:**
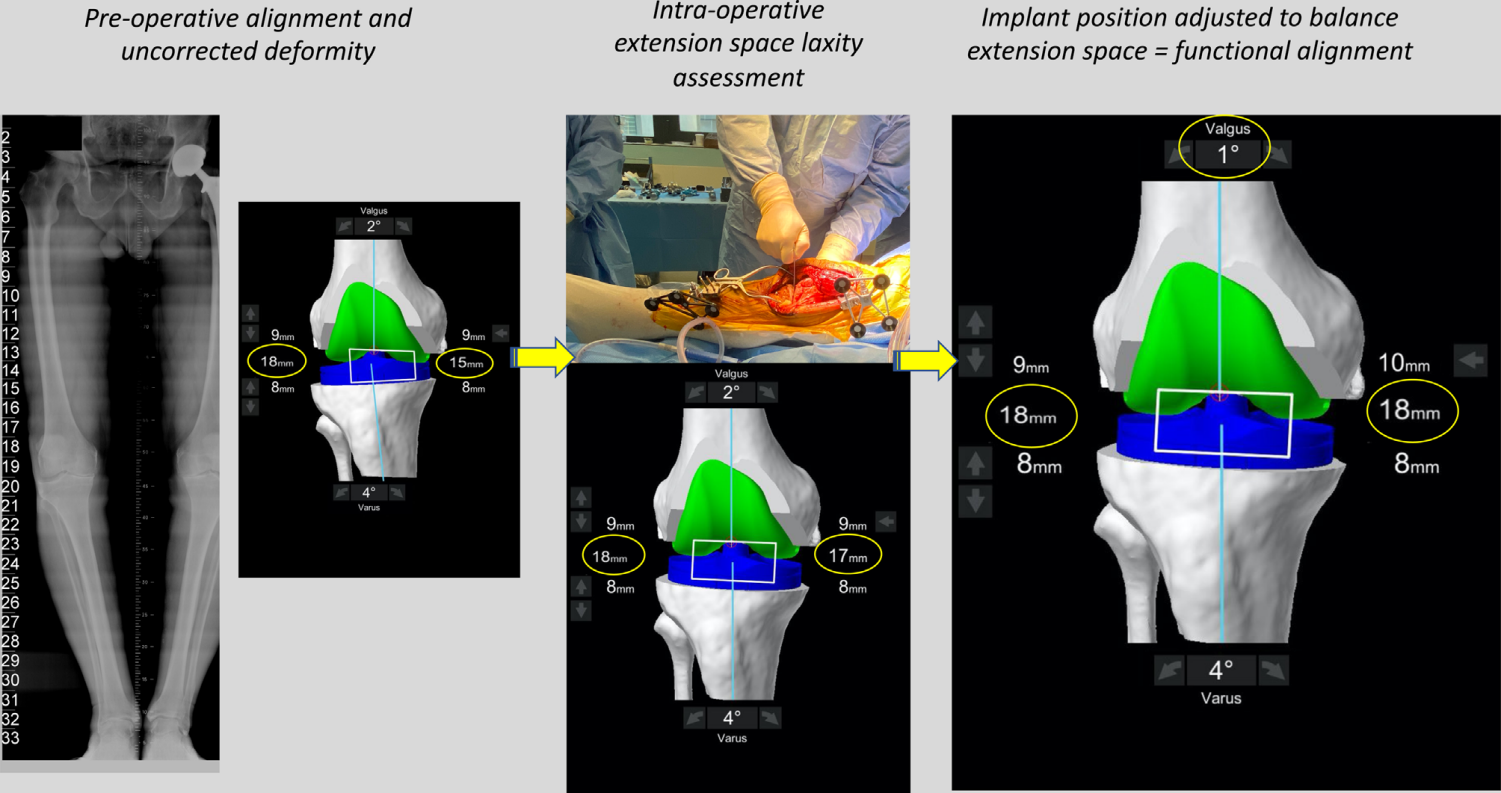
Intra-operative assessment of the extension space. The limb is placed in a corrected position, and the robot “captures” the pose. The personalized plan will deliver extension gaps of 18 mm laterally and 17 mm medially. In order to achieve balanced compartments, the plan is modified in this case by decreasing the femoral valgus.

**Figure 5:**
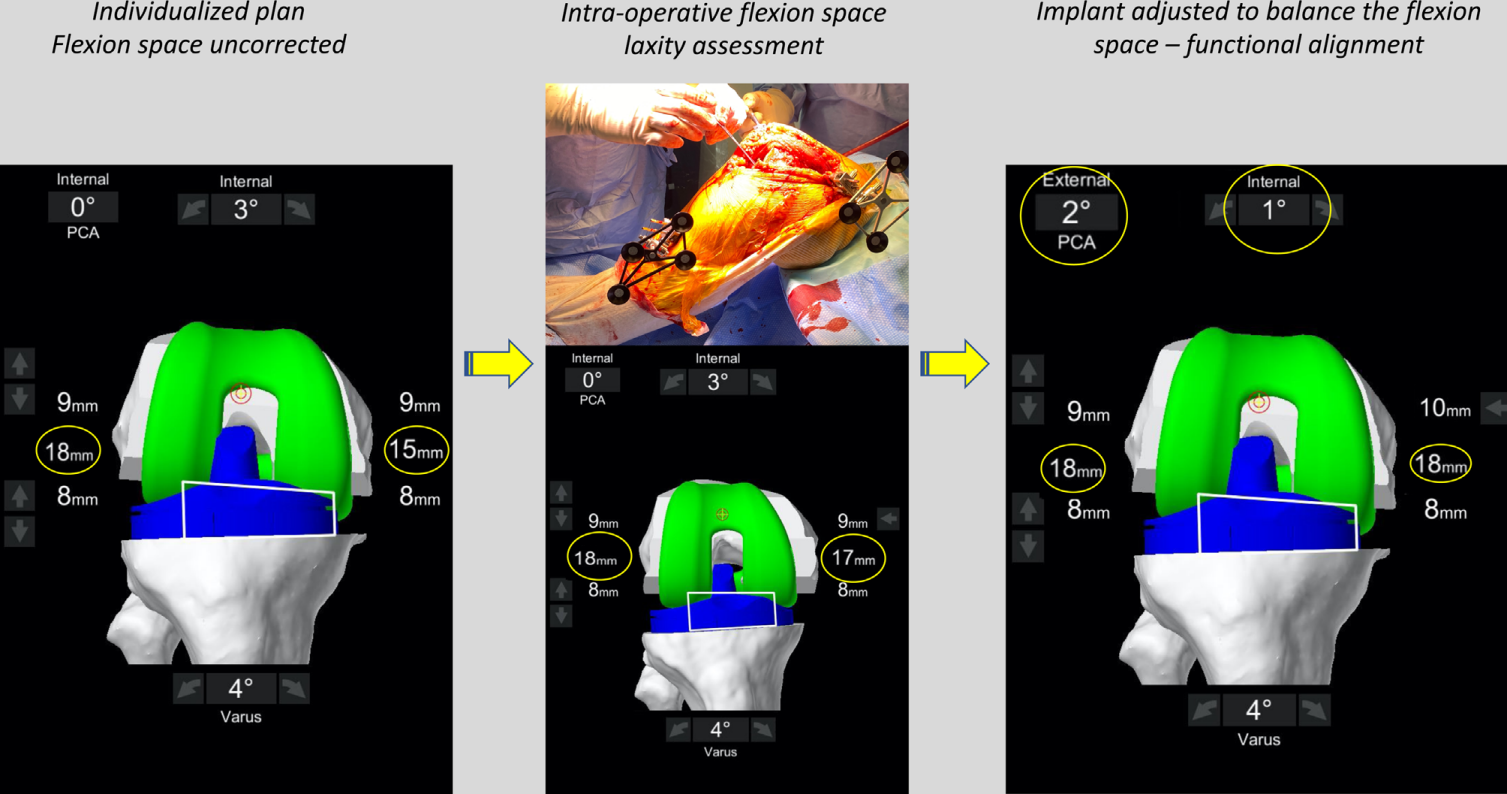
The flexion space before correction. The flexion space is assessed using sized spacer spoons until the corrected position is achieved. The personalized plan will deliver a medial space of 17 mm and lateral space of 18 mm. In order to balance the flexion space, the implant is externally rotated until the compartments are balanced.

The functional philosophy aims to reconstitute native alignment, but in addition, will consider the contribution of the soft tissue envelope (Figures 4 and 5). *The target coronal alignment guide is set within the limits of 174° (6° varus) to 180° for a varus knee.* In our experience, 95% of varus aligned primary TKA cases will have a constitutional HKA between 174° and 180°.

Evidence continues to emerge regarding target coronal alignment. A natural variation in HKA exists from 170° to 183° in native non-osteoarthritic knees [21]. Hirschmann et al. has also demonstrated significant variability in gender differences in normal alignment, with 15.0% of females observed to have an HKA angle between 175.5° and 178.5° compared to 29.2% of males. Furthermore, 10.8% and 4.4% of males and females, respectively, had an HKA between 172.5° and 175.5° [22]. Safe zones that set a limit of ±3° do not allow constitutional alignment to be recreated in at least 20% of the population [18, 23]. Several studies have demonstrated good clinical results out to 10-year follow-ups outside this range [20, 24–28]. Specifically, residual varus alignment up to 6° varus is an approved boundary of the US Food and Drug Administration and has been shown to have good 10-year implant survival (no failures) [28] without evidence of migration [29].

#### c. Implant adjustments to respect joint line-height and obliquity

Functional alignment aims to preserve JLO and joint line-height. JLO is maintained through orientation of the distal femoral and proximal tibial bone using the arithmetic hip-knee-angle (aHKA) method described by Macdessi et al. [30]. Briefly, it is calculated by subtracting the LDFA from the MPTA. If the aHKA is negative, the constitutional alignment is varus, and valgus if the aHKA is positive*.* Recently we reported our results on over 1000 primary TKA, demonstrating improved pain scores when native “apex distal” JLO was maintained. Furthermore, 98.9% of the population presenting for TKA had an obliquity of either <177° (63.5%) or 177°–183° (35.4%), and a MA philosophy maintained the native JLO in only 18% of cases [13]. Therefore, *a key goal of FA is to maintain the JLO phenotype of the patient.*

Raising the joint line may result in mid-flexion instability [31, 32]. One of the concerns regarding the KA and rKA is that balancing is often achieved by re-cutting the tibia, with increasingly thicker polyethylene inserts being required. We have previously demonstrated that this approach for balancing leads to increased valgus laxity through an arc of flexion [33]. *For this reason, in FA, the maximum alteration to joint line-height is set within the limits of ±3 mm*, and when large gaps are present (for example, 21 mm), we adjust the depth of the cuts through a combination of femoral and tibial alignment adjustment.

#### d. Equal laxity of tibio-femoral compartments in flexion and extension

Pre-emptive soft-tissue balancing is a critical step of FA. *The aim is to achieve flexion and extension gaps goals in the medial and lateral compartments that are equal to the global thickness of the implant* (which is 17.5 mm). A slight lateral laxity (1 mm more than in the medial compartment) is acceptable for the varus aligned knee. In simple terms, this means finishing with gaps in the tibiofemoral compartments (medial/lateral) that are equal to or less than 1.5 mm of each other. Additionally, the final gaps in full extension and 90° flexion should not be more than 2 mm from the global implant thickness (i.e., 19 mm for a 17 mm thickness).

Several robotic platforms can predict gaps with adjustments to the implant position pre-emptively before any bone cuts are made. This step represents a major departure from kinematic philosophies that aim for a measured resection [5, 34]. In FA, balanced laxity is achieved prior to cuts being made by placing the implant in a position that fits the behavior of the patient’s knee through an arc of flexion. In our experience, this requires changing the rotation of the posterior femoral cut within a range of 3° from the posterior condylar axis (PCA) or 6° from the transepicondylar axis (TEA) in 90% of cases to achieve balance in flexion. A method for accurately quantifying laxity is described in the surgical technique section.

#### e. Implant Sizing to bony anatomy

Modern implant designs do not account for the wide variety of distal femoral anatomy seen in the normal population [35, 36]. Furthermore, the variability between AP and mediolateral dimensions in the femur or the medial and lateral tibial plateaus may lead to the imperfect matching of implant sizing to the patient’s anatomy [37].

##### Femur

The femoral component size is first matched around the radius of curvature of the distal femur, recreating the native depth of the trochlear groove. The position is adjusted to ensure the bone cuts exit the anterior femoral cortex without notching. Femoral flexion is set within a 0–10° threshold in relation to the femoral mechanical axis (FMA), and combined flexion (tibial slope + femoral flexion) should avoid exceeding 10° for this implant. Second, the mediolateral fit of the component is checked, and the implant position is laterally translated while avoiding any component overhang. Personalized planning also allows the surgeon to preoperatively pre-empt matching problems that may arise between the implant size and the anatomy.

##### Tibia

The tibia is initially sized with rotation to 0° on the axial view of the CT scan with the aim of having maximal cortical contact with no implant overhang. The tibial slope is matched to the medial tibial plateau, within a 0–3° limit when using a PS implant, combined flexion (tibial slope + femoral flexion) should avoid exceeding 10°. Rotation is set manually.

#### f. Final limb sagittal alignment to achieve full extension

Sagittal limb alignment refers to the position of the knee as measured by the robotic system with only the force of gravity, i.e. when the foot is held off the table with the patella in the reduced position. *One of the surgical goals in FA is the correction of the sagittal deformity (0° extension under gravity).* The precise effect of residual sagittal limb alignment in TKA remains unclear. While satisfaction and PROM’s appear to be improved when a fixed-flexion-deformity is eliminated [38], this effect does not appear to be observed when the residual position is less than 5° from neutral [39]. We have recently demonstrated that a residual recurvatum deformity of 5° can be well tolerated at 5-year follow-up using a PS implant in patients who initially had a recurvatum deformity [40]. *For this reason, we aim for the final limb alignment to be no more than 5° from neutral following implantation of final components. The combined sagittal alignment of the implants (tibial slope and femur flexion) should also avoid exceeding 10° with this implant.*

### II Functional Alignment Workflow

**Figure 6:**
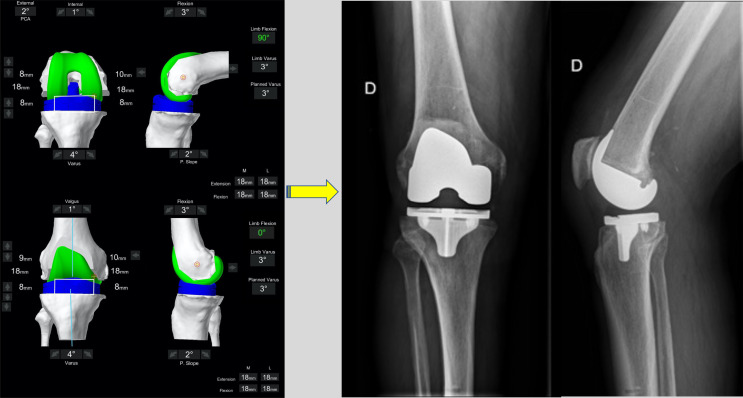
The final intra-operative position. Limb alignment, resections depth, and tibiofemoral gaps in flexion and extension are shown. The achieved coronal alignment is 177° or 3° varus, and the sagittal alignment is 0° of flexion. The post-operative X-ray at 3 months follow-up of the same patient is shown.

Images illustrating the key sequential steps for FA of a typical varus morphotype knee are summarised in Figures 3–6. FA requires a robotic platform that provides real-time 3D feedback to the surgeon on the implant position and limb alignment as well as virtual flexion and extension gaps. The following description is using the MAKO robotic platform (Stryker, Kalamazoo, MI, USA) with a Triathlon PS constrained implant. The thinnest combined thickness of the implants (9 mm polyethylene) is 17.5 mm. The MAKO robotic platform is an image-based system utilizing a pre-operative CT scan to create a 3D model of the patient’s bony anatomy. This system has been demonstrated to be more accurate than a manual technique [16], cause less soft tissue damage [41], and deliver the intended plan with an accuracy of approximately 1° [17].

#### Surgery

- *Individualized pre-operative planning*

An individualized plan is created from the pre-operative CT scan and X-rays as outlined in the principal’s section (Figure 3).

- *Anatomy Registration and ligament balancing*

The MAKO set-up and landmarks registration are performed as previously described [42]. Attention should be paid to removing osteophytes, and adhesions, especially those that are commonly found attenuating the collateral ligaments. Assessment of ligament balancing is performed after registration of the femoral and tibial landmarks and before any bone resection. Retractors must be removed from the surgical field at this point and the patella in the reduced position. A complete sequence demonstrating the technique for soft tissue assessment in the varus knee is demonstrated in Figures 4 and 5.

Two positions are “captured” by the robotic system, the first in extension (Figure 4) and the second in flexion (Figure 5). The effect of the planned bony cuts and implant positioning on the captured joint positions is displayed on the computer interface allowing pre-emptive changes to be made based on desired targets for alignment and tibiofemoral gaps. The robot displays the combined thickness of the implant required with the pre-operative plan to achieve the “captured” or target soft tissue gap set by the surgeon. While assessing laxity in extension manually is technically easy, flexion laxity is more challenging as contributions from the thigh and hip are not eliminated. For this reason, we assess gaps using a spoon (Figures 4 and 5). Spacer spoons are inserted into the worn compartment. *In order to avoid over or under correction of the joint line, the spacer spoon should open the compartment without causing changes to the contralateral tibiofemoral space.* For example, in a varus knee, spoons are inserted into the medial compartment until gap changes begin in the lateral compartment. This can be assessed by visually inspecting the compartment and by feedback from the robotic navigation screen. In some circumstances, it may be necessary to place spoons into both compartments to correctly restore the joint line and tension of the ligaments due to a large degree of medial and lateral laxity. In our experience, this was seen in patients with a significant recurvatum deformity or varus thrust.

Following this, the virtual 3D plan is modified by dynamic gap balancing to adjust the implant position so that a combined thickness of 17 mm in flexion and extension is required in the lateral and medial compartments (global thickness of the implant). However, some adaptations in the space gaps may be necessary for specific pre-operative situations. Indeed, targets of 16 mm in cases of hyperextension (recurvatum) and 18 mm for fixed-flexion deformity are aimed for the extension gap.

- *Intra-Operative Planning*

The surgeon can manipulate the implant positions in a variety of ways in order to achieve tibiofemoral balancing. However, any adjustments must be made with the principles of respecting joint line-height and orientation. A systematic approach is required to balance so that the difference between medial and lateral gaps is not greater than 1 mm in flexion and extension.

1. Extension space balancing

This space is most profoundly affected by the coronal positioning of the femoral and tibial components. Adjustments are made in the femoral and tibial coronal position within the confines of 3 mm variation from the native joint line-height. The JLO phenotype should also be maintained, and an overall HKA does not result in a non-constitutional alignment (i.e., previously varus knee aligned into valgus alignment).

1. Flexion space balancing

The flexion space asymmetry is mostly affected by adjustments to femoral rotation. In the varus knee, the femoral rotation is adjusted around a medial pivot point centered on the medial femoral condyle. While initial plans are set to match the PCA, adjustments are made as required to balance this space. We use a limit of 3° freedom from the PCA, or up to 6° ER to 3° IR from the TEA for femoral rotation. The current US FDA limit for rotation is set at 6° freedom from the TEA. The internal rotation beyond 3° from the TEA should be avoided in FA. Furthermore, as the femoral cuts become more externally rotated, the likelihood of notching increases, and to avoid this, our preference is to increase the femoral component flexion. Upsizing the femur increases the likelihood of implant overhang, anteriorising the femoral component increases the flexion space and may also overstuff the patellofemoral joint.

## Discussion

The aim of this paper was to describe the FA philosophy in TKA for the varus morphotype using a robotic platform. FA is a philosophy that utilizes recent advancements in robotically assisted knee arthroplasty to encompass recreating constitutional alignment, joint line height and obliquity with adjustments to the implant positions being made based on a quantifiable soft tissue laxity assessment through an arc of flexion. Furthermore, with the use of a robotic platform, errors in the final position can be pre-empted prior to any bone cuts being made, avoiding the need for soft tissue releases, and the delivery of the final plan is done with a high degree of accuracy [16, 17]. Finally, FA considers the behavior of varus and valgus morphotypes to be significantly different, requiring a different approach for each.

Alignment techniques have tended to focus on coronal alignment with factors such as joint line-height, gap balancing, and JLO not being controlled for. Early alignment philosophies aimed for a neutral HKA for all patients and relied on releasing soft tissues to achieve balancing [1, 7]. The KA philosophy was based on bony landmarks and cartilage wear, without safe zones for implant positioning [5]. Only inverse KA considers defined endpoints for joint laxity but achieves balancing through adjustment of the femoral component. These previous strategies have not defined targets for JLO or height, and the assessment of soft tissue laxity has not been objective, making it difficult to reproduce techniques. Functional alignment considers safe zones for alignment goals but aims for definable and reproducible soft tissue targets. Furthermore, it avoids changing JLO phenotypes, considers the height of the joint line, and has strict rules regarding implant sizing. This philosophy utilizes the considerable recent developments in understanding native knee joint behavior and surgical tools to aim for objectively definable and reproducible goals beyond that of coronal joint alignment.

*The major difference of the FA to previous philosophies is the modification of alignment based on a combination of constitutional bony anatomy and soft-tissue laxity assessment.* It is established that instability after knee arthroplasty is a cause for failure [10, 43] (particularly in younger patients) [44], and there is emerging evidence demonstrating that patient pain, and satisfaction scores are related to achieving a target range of tibiofemoral gaps intra-operatively [12, 45–47]. Wakelin et al. used a robotic platform to demonstrate when gap thresholds of an equally balanced compartment or mediolateral imbalance of less than 1.5 mm were achieved with a PS implant, it was associated with significantly improved KOOS pain scores at one-year follow-up [12]. Criteria for achieving balance are not yet well defined and may differ based on prosthesis design, i.e., Medial-pivot versus PS, CR constrained designs, or single versus multi-radius curvature femoral components. A wide variation in natural laxity of the collateral ligaments has also previously been described [34] in non-arthritic knees and between genders [48]. The behavior of the ligaments also differs in flexion from that of an extension [34]. Furthermore, osteophytes have also been shown to significantly alter the laxity of the MCL in extension and 30° of flexion [49]. With further research, target boundaries will become more evident, and it is likely that a gap-targets will become personalised to patients.

The defined target safe zones for the final HKA are not yet resolved. Mechanical alignment aimed for a final HKA of 180°, however when reported in the literature, the vast majority of studies consider any knee arthroplasty to be within 3° of 180° to be MA. Kinematic alignment did not set safe zones, and due to concerns regarding extreme implant positions, several authors have attempted to define boundaries [4, 6, 50]. Setting safe zones within 3° of a neutral MA is restrictive and recreates the same problem that saw alternative philosophies to MA being explored originally.

Currently, FA safe zones are set from 6° varus to 0° valgus for final HKA and tibial coronal alignment and 6° valgus to 3° varus for femoral coronal alignment and ±6° to the surgical TEA for femoral rotation for varus knees. These are also the current approved limits set by the US food and drug administration, and Bellemans et al. demonstrated 90% of native knees fall within this range [51]. The native knee is said to bear more load through the medial tibiofemoral compartment in the varus knee [1], favoring a varus position, and this has been demonstrated to tolerate post-TKA up to 7° from neutral without compromising implant survival or loosening with the prosthesis described in this paper [28, 52]. Growing evidence regarding safe limits of implant positioning continues to emerge, challenging the concerns that exist about the risk of early failure of components implanted outside of a neutral MA [1, 53]. A recent RCT demonstrated no migration or increased risk of lucency when having an overall HKA angle was in the range of 6° varus to 2° valgus. Furthermore, the tibial component could be placed in the range of 7° varus to 2° valgus without the risk of component migration.

### Future study and weakness’s

Several questions remain unanswered. First of all, this technique assumes that balancing in extension and then at 90° flexion results in a balanced knee throughout a range of motion, which makes sense with a single-radius implant. The ability to plan and have feedback through a greater range would be an advancement to the current description. Furthermore, this describes a goal of achieving equal gaps in flexion and extension. It has previously been described that the native knee has more laxity in flexion than an extension and laterally more than medially. Further research is required to identify the optimal gap target, with tailoring soft tissue tension to individual patients being a possibility. Finally, concerns regarding safe limits for implant positioning remain, with thresholds being poorly defined and with little evidence to support rigid limits. Ultimately the answer to this question will only be realized with long-term follow-up data, which is yet to come.

## Conclusion

Functional alignment is a novel alignment technique that aims to restore constitutional bony alignment and balance the laxity of the soft tissues by placing and sizing implants in a manner that respects the variations in individual anatomy. This paper presents our current approach for the varus morphotype.

## Conflict of interest

JS, CB, ESM: declare that they have no conflict of interest.

ES: Consultant for Corin.

SL: Royalties from Smith Nephew and Stryker. Consultant for Stryker, Smith Nephew, Heraeus, Depuy Synthes, Groupe Lepine; Institutional research support from Corin, Amplitude; Editorial Board for Journal of Bone and Joint Surgery (Am).

## Funding

No funding was received for this study.

## Ethics approval

Ethical approval was not required.

## Authors’ contributions

JS: Manuscript writing, editing, conceptualization and visualization.

CB: Manuscript writing, conceptualization, and visualization.

ES: Manuscript writing, conceptualization, and visualization.

SG: Manuscript writing, conceptualization, and visualization.

SL: Conceptualization, original manuscript writing, editing.
