# Correction Notice to: Functional Alignment Philosophy in Total Knee Arthroplasty – Rationale and technique for the varus morphotype using a CT based robotic platform and individualized planning

**DOI:** 10.1051/sicotj/2022017

**Published:** 2022-05-20

**Authors:** Jobe Shatrov, Cécile Batailler, Elliot Sappey-Marinier, Stanislas Gunst, Elvire Servien, Sebastien Lustig

**Affiliations:** 1 Department of Orthopaedics, Croix Rousse Hospital, University of Lyon 1 69004 Lyon France; 2 Sydney Orthopaedic Research Institute Chatswood 2065 Australia; 3 Univ Lyon, Claude Bernard Lyon 1 University, IFSTTAR, LBMC UMR_T9406 69622 Lyon France

The article was published April 1st, 2022 with an error in the first name of the second co-author.

The full name of the second author is: Cécile Batailler.

